# Association of Serum Galectin-3 with Aortic Stiffness in Adults with Stage 3–5 Non-Dialysis Chronic Kidney Disease: Overall and Stage-Stratified Analyses

**DOI:** 10.3390/biomedicines14102213

**Published:** 2026-09-30

**Authors:** Yahn-Bor Chern, Ho-Hsiang Chang, Yu-Li Lin, Hung-Hsiang Liou, Jen-Pi Tsai, Bang-Gee Hsu

**Affiliations:** 1 Division of Nephrology, Department of Internal Medicine, Yuan’s General Hospital, Kaohsiung 802635, Taiwan; 2 Division of Nephrology, Hualien Tzu Chi Hospital, Buddhist Tzu Chi Medical Foundation, Hualien 970473, Taiwan; 3 School of Medicine, Tzu Chi University, Hualien 970374, Taiwan; 4 Division of Nephrology, Department of Internal Medicine, Hsin-Jen Hospital, New Taipei City 242009, Taiwan; 5 Division of Nephrology, Department of Internal Medicine, Dalin Tzu Chi Hospital, Buddhist Tzu Chi Medical Foundation, Chiayi 622401, Taiwan

**Keywords:** chronic kidney disease, galectin-3, aortic stiffness, carotid-femoral pulse wave velocity

## Abstract

**Background/Objectives:** Cardiovascular injury is common in chronic kidney disease (CKD), but the relationship between serum galectin-3 and aortic stiffness (AS) in adults with non-dialysis CKD remains unclear. This study evaluated the association between serum galectin-3 and AS in adults with stage 3–5 non-dialysis CKD. **Methods:** This cross-sectional study included 176 adults with stage 3–5 non-dialysis CKD. Serum galectin-3 concentrations were quantified using an enzyme-linked immunosorbent as-say, and carotid-femoral pulse wave velocity (cfPWV) was measured to assess AS, de-fined as cfPWV > 10 m/s. **Results:** Sixty-three participants (35.8%) met the criterion for AS. Compared with the non-AS group, participants with AS were older and had a higher prevalence of diabetes mellitus, higher systolic blood pressure (SBP), fasting glucose, urine protein-to-creatinine ratio, and serum galectin-3 levels. Multivariable logistic regression analysis demonstrated that galectin-3 (odds ratio [OR], 1.100; 95% confidence interval [CI], 1.015–1.192; *p* = 0.020), age (OR, 1.068; 95% CI, 1.026–1.111; *p* = 0.001), and SBP (OR, 1.158; 95% CI, 1.098–1.221; *p* < 0.001) were independently associated with AS. After adjustment for covariates, log-transformed galectin-3 remained independently associated with cfPWV in stepwise linear regression. **Conclusions:** Higher serum galectin-3 concentrations were independently associated with AS in adults with stage 3–5 non-dialysis CKD.

## 1. Introduction

Chronic kidney disease (CKD) is associated with a significant increase in cardiovascular risk prior to the initiation of kidney replacement therapy [1]. Among cardiovascular complications, aortic stiffness (AS) is of particular clinical importance because it reflects structural and functional damage in large arteries and imposes an excessive pulsatile load on “end organs,” including the heart, brain, and kidneys [2,3,4,5].

In CKD, large-vessel injury is not limited to atherosclerotic plaque burden. It also involves arteriosclerosis, medial vascular calcification, and impaired compliance of the central arteries [6,7,8]. Ongoing inflammation, increased oxidative stress, dysfunction of the vascular endothelium, accumulation of uremic toxins, and imbalances in mineral metabolism can work together to accelerate these structural changes, leading to a gradual increase in aortic wall stiffness [7,8,9]. As arterial stiffness increases, pulse waves travel more rapidly and return earlier, thereby increasing left ventricular afterload, widening pulse pressure, reducing diastolic coronary perfusion, and transmitting excessive pulsatile stress to the cerebral and renal circulation [9,10]. These hemodynamic alterations provide a pathophysiological basis for the high burden of macroangiopathic complications in CKD and support the clinical relevance of assessing AS in this population [2,7,11].

Given this pathophysiological background, carotid–femoral pulse wave velocity (cfPWV) is considered the standard noninvasive method for measuring AS, both in clinical practice and in research [10,12,13]. When pulse wave velocity (PWV) is high, it indicates a greater risk of cardiovascular events and all-cause mortality, regardless of population. Recent studies show that PWV provides incremental prognostic information beyond traditional cardiovascular risk factors [14,15]. In CKD patients, arterial stiffness and worsening kidney function may coexist, as both may be influenced by chronic inflammation, vascular injury, mineral metabolism disturbances, oxidative stress, and sustained vascular stress [6,16]. Galectin-3 is a protein implicated in fibrosis, myocardial remodeling, and inflammation, which has led researchers to propose its role as a potential mediator of cardiovascular disease [17,18]. Earlier studies have reported that higher renal or circulating galectin-3 levels are associated with lower kidney function (as measured by estimated glomerular filtration rate [eGFR]) and greater inflammation, as indicated by higher C-reactive protein levels. This supports a link between galectin-3, fibrosis, and inflammation [19,20,21]. Although higher galectin-3 levels in patients with CKD have been associated with adverse clinical outcomes, the available evidence remains inconsistent [22]. More broadly, cardiovascular risk stratification in CKD is unlikely to rely on a single circulating biomarker. Provenzano et al. reviewed prognostic and predictive biomarkers spanning kidney function, cardiac injury, inflammation, fibrosis, endothelial dysfunction, and vascular remodeling, and concluded that although these markers are pathophysiologically informative, most still require stronger validation before routine clinical implementation [23].

Although previous studies have linked circulating galectin-3 to aortic stiffness in a community sample [24], vascular dysfunction in patients with stage 3–5 CKD [19], and cfPWV-defined AS in dialysis patients [25], evidence specifically examining cfPWV-defined AS in adults with stage 3–5 non-dialysis CKD remains limited. The present study therefore adds information by evaluating whether serum galectin-3 is associated with AS in established non-dialysis CKD after accounting for conventional cardiovascular and renal risk factors, including age, SBP, diabetes-related variables, UPCR, and eGFR. We also explored whether this association differed across CKD stages.

## 2. Materials and Methods

### 2.1. Study Population and Eligibility

This cross-sectional study was conducted in 176 adults with stage 3–5 CKD who were recruited from the nephrology outpatient clinic of a medical center in Hualien, Taiwan, from 1 May to 30 August 2023. Eligible patients were consecutively invited to participate during the recruitment period, and those who provided written informed consent were enrolled. The enrollment criteria for this study were that all participants be adults aged 18 years or older and not receiving dialysis at the time of enrollment. None of the patients with stage 5 CKD were enrolled if there was a clinical indication to initiate dialysis (e.g., volume overload, life-limiting hyperkalemia or metabolic acidosis, or overt uremic manifestations), in line with current guideline-based practice [1]. With regard to the definition of CKD, two eGFR measurements of <60 mL/min/1.73 m^2^, separated by 3 months, defined CKD, and eGFR was calculated with the 2021 CKD-EPI equation. CKD stages 3, 4, and 5 were defined by eGFR ranges of 30–59, 15–29, and <15 mL/min/1.73 m^2^, respectively, according to Kidney Disease Outcomes Quality Initiative criteria [1]. During the study period, participants continued to receive standard multidisciplinary CKD care, including dietary counseling and recommendations to avoid nephrotoxic agents. Detailed inclusion and exclusion criteria are presented in Table 1.

**Figure 1:**
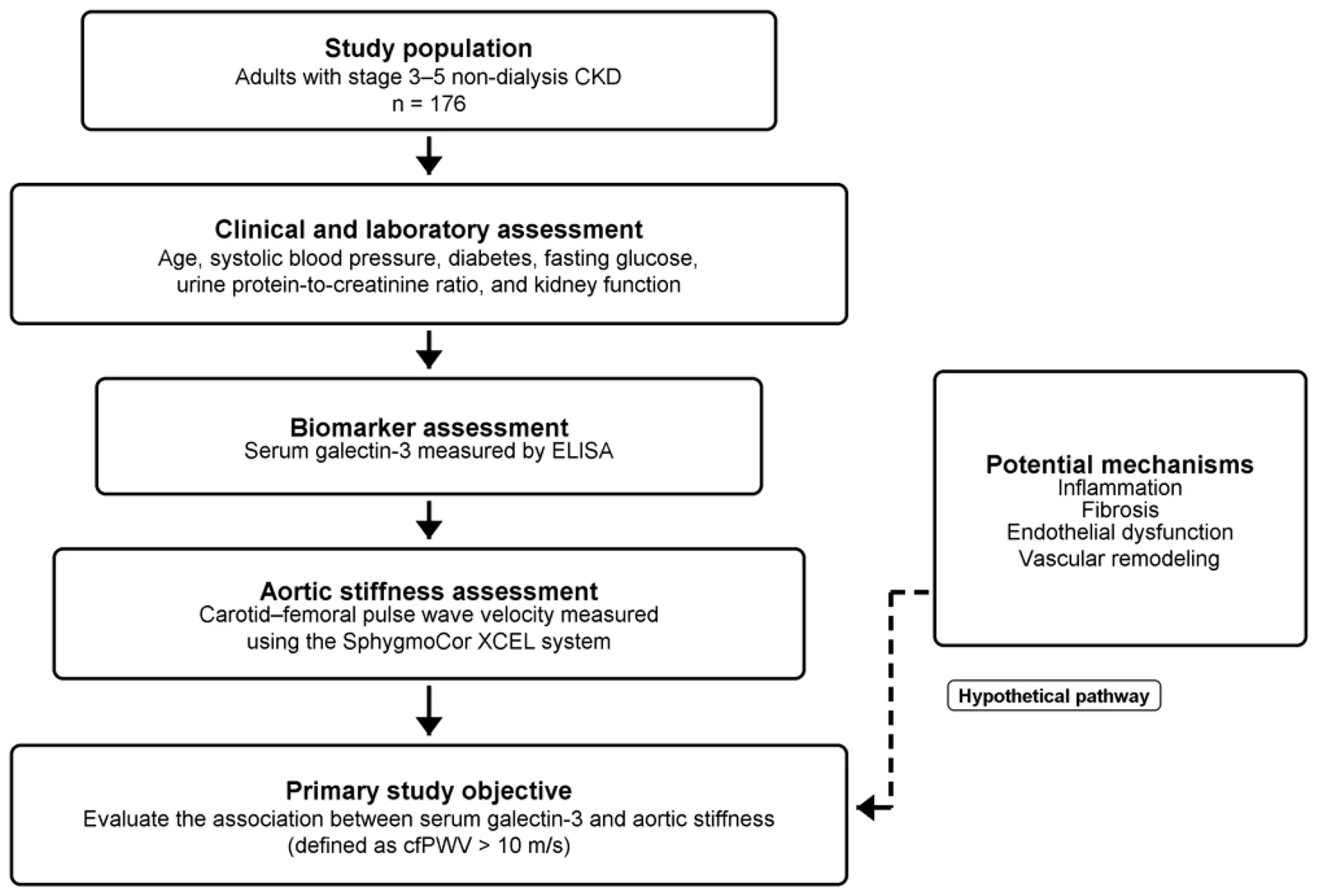
Schematic overview of the study design and the hypothesized mechanisms linking galectin-3 to aortic stiffness in adults with stage 3–5 non-dialysis chronic kidney disease. Clinical and laboratory variables, serum galectin-3, and carotid-femoral pulse wave velocity were assessed, and aortic stiffness was defined as carotid-femoral pulse wave velocity greater than 10 m/s. Proposed mechanisms linking galectin-3 to aortic stiffness include inflammation, fibrosis, endothelial dysfunction, and vascular remodeling.

Clinical comorbidities were based on both participant interviews and medical record reviews. The case definition for diabetes mellitus was a fasting plasma glucose level of 126 mg/dL or more, or use of glucose-lowering medication. Hypertension was defined as systolic blood pressure (SBP) ≥ 140 mmHg, diastolic blood pressure (DBP) ≥ 90 mmHg, or current antihypertensive treatment. Ethics approval was given by the Research Ethics Committee of Hualien Tzu Chi Hospital, Buddhist Tzu Chi Medical Foundation (IRB108–219-A). The investigation adhered to the Declaration of Helsinki, and written informed consent was obtained from each participant prior to beginning the study. Figure 1 provides a schematic overview of the study design and the hypothesized mechanisms linking galectin-3 to AS in non-dialysis CKD.

### 2.2. Body Measurements for Anthropometric Information

Anthropometric data were collected at the study visit by trained staff. Participants were weighed and their standing height was recorded while they wore light clothing and no shoes. Body mass index was then obtained by dividing body weight by height squared (kg/m^2^). Before vascular assessment, seated brachial SBP and DBP were measured as the average of three measurements using an automated sphygmomanometer after a minimum of 30 min of rest in the seated position.

### 2.3. Sample Collection and Laboratory Procedures

Venous blood was taken at the morning visit following a fast of >8 h overnight, with 5 mL collected per subject. Immediately following venipuncture, specimens were centrifuged (3000× *g* for 10 min). Serum was separated within 1 h and stored at 4 °C until analysis. Hemoglobin was measured using an automated hematology analyzer (Sysmex XS-1000i; Sysmex America, Mundelein, IL, USA). Routine biochemical testing was performed using an Advia 1800 autoanalyzer (Siemens Healthcare GmbH, Henkestr, Germany). Renal indices were blood urea nitrogen (BUN) and creatinine. Additional variables were measured: fasting glucose and albumin. Lipid profile included total cholesterol, triglycerides, and low-density lipoprotein-cholesterol (LDL-C). Serum galectin-3 levels were measured by enzyme-linked immunosorbent assay kits from RayBiotech (Peachtree Corners, GA, USA). A spot urine sample was also collected to determine the urine protein-to-creatinine ratio (UPCR).

### 2.4. Aortic Stiffness Measurement

AS was evaluated by cfPWV using the SphygmoCor XCEL system (AtCor Medical, Sydney, Australia). Before the measurement procedure, participants remained seated for 30 min. Brachial blood pressure was then measured three times at 5-min intervals, and the mean value was used in the subsequent analysis. Each of these cfPWV assessments was conducted in the morning. The measurements were taken in a quiet room with controlled temperature. Brachial SBP and DBP were measured oscillometrically with an XCEL cuff on the left upper arm. The cuff was then reinflated to a subdiastolic pressure level. Simultaneous measurements of femoral volume-displacement waveforms were acquired in conjunction with a cuff applied to the upper thigh. Due to the standard operating procedure, cfPWV was produced automatically by SphygmoCor XCEL software (version 1.30).

In accordance with guideline recommendations, participants with cfPWV values above 10 m/s were classified as having AS, whereas those with values of 10 m/s or below were assigned to the non-AS group [10].

### 2.5. Analytical Strategy

All analyses were carried out using version 25.0 of SPSS for Windows (IBM Corp, Armonk, NY, USA), as well as R v4.2.2 (R Foundation for Statistical Computing, Vienna, Austria). Throughout the study, we performed statistical inference using two-sided tests. A *p*-value of <0.05 was considered statistically significant.

A sample size consideration was performed for the primary association analysis between serum galectin-3 and cfPWV. Based on the method described by Hulley et al. [26], a minimum of 85 participants was required to detect a correlation coefficient of 0.30 with a two-sided alpha level of 0.05 and 80% power. Therefore, the final cohort of 176 participants was considered sufficient for the overall association analysis. However, the CKD stage-stratified analyses, particularly the stage 5 subgroup, were considered exploratory because of the smaller number of participants in each stratum.

Data were summarized by variable type and distributional pattern. Continuous variables are presented as mean ± standard deviation or median (interquartile range), as appropriate. For categorical variables, counts and percentages are provided. Characteristics of data guided selection of between-group comparisons that were performed with independent Student’s *t*-test, Mann–Whitney *U* test or chi-square tests. Variables showing skewed distributions were analyzed after logarithmic transformation. Before model fitting, descriptive statistics and distributional patterns were reviewed to identify implausible values and extreme observations. No observations were excluded solely on the basis of being statistical outliers when no data-entry or measurement error was identified. Skewed continuous variables were handled by logarithmic transformation, as described above. Complete data were available for all 176 participants for every variable reported in Table 2 and included in the statistical analyses, with 0 missing observations for each variable. No participants were excluded because of missing data, and no imputation was performed. Variables showing significant differences between the AS and non-AS groups (*p* < 0.05) were included in the multivariable logistic regression model. DM and fasting glucose were retained as separate covariates because DM reflects established disease status and/or glucose-lowering treatment, whereas fasting glucose represents current fasting glycemic status at the time of blood sampling. Their simultaneous inclusion allowed us to assess whether the association between galectin-3 and AS was independent of both clinical diabetes status and current fasting glycemia. An additional sensitivity analysis was conducted by adding eGFR as a continuous covariate to the original model to evaluate the association between serum galectin-3 and AS after further adjustment for kidney function. eGFR was included on the basis of its clinical relevance, irrespective of its between-group *p*-value, and all original predictors were retained. Multicollinearity among the covariates was assessed using tolerance and variance inflation factor values. To examine the determinants of cfPWV as a continuous outcome, we constructed a multivariable forward-stepwise linear regression model. We also repeated the analyses after stratifying the cohort by CKD stage to assess whether the association between galectin-3 and cfPWV differed across stages 3, 4, and 5. Functional forms of continuous predictors were assessed in supplementary analyses using restricted cubic splines with three knots at the 10th, 50th, and 90th percentiles of each predictor on its modeled scale. For each predictor separately, one nonlinear spline component was added while retaining the corresponding linear term and all other covariates. Nonlinearity was evaluated using nested likelihood-ratio tests for logistic regression and partial F tests for linear regression, each with one additional degree of freedom. Log-transformed predictors were assessed on the logarithmic scale. Diagnostics were performed for the original models, the eGFR-adjusted logistic model, and a cfPWV model retaining age, SBP, ln(fasting glucose), eGFR, ln(UPCR), and ln(galectin-3). Additional exploratory models simultaneously incorporated spline terms for galectin-3, SBP, eGFR, and UPCR, using the corresponding modeled scales.

Spearman’s rank correlation analysis was used to evaluate associations between galectin-3 and clinical variables.

## 3. Results

### 3.1. Baseline Characteristics

A total of 176 patients with nondialysis CKD were included in the analysis. Among them, 63 patients (35.8% of the total) had AS, whereas 113 (64.2%) did not. The distribution of CKD stages in the overall cohort was 39.8% for stage 3 (*n* = 70), 34.7% for stage 4 (*n* = 61), and 25.6% for stage 5 (*n* = 45). The principal underlying causes of CKD were diabetic kidney disease in 67 patients (38.1%), glomerulonephritis in 46 patients (26.1%), and hypertensive nephrosclerosis in 25 patients (14.2%).

Patients with AS were significantly older than those without AS (*p* < 0.001). In the AS group, SBP (*p* < 0.001), fasting glucose (*p* < 0.001), UPCR (*p* = 0.006), and serum galectin-3 (*p* = 0.002) were all significantly higher than in the non-AS group. Diabetes mellitus was also more prevalent in the AS group than in the non-AS group (*p* < 0.001).

No statistically significant differences were observed between groups in sex, height, body weight, body mass index, DBP, hemoglobin, total cholesterol level, triglycerides, LDL-C, BUN, creatinine, eGFR, prevalence of hypertension or glomerulonephritis subtype, or use of angiotensin receptor blockers, beta-blockers, calcium channel blockers, statins, or fibrates. CKD stage distribution was also similar between groups (all *p* > 0.05), as shown in Table 2.

### 3.2. Factors Independently Associated with Aortic Stiffness

Variables that differed significantly between the AS and non-AS groups in Table 2, including age, SBP, diabetes mellitus, fasting glucose, UPCR, and serum galectin-3, were entered into the multivariable logistic regression model. After adjustment for potential confounders, serum galectin-3 levels remained independently associated with AS (odds ratio [OR]: 1.100; 95% confidence interval [CI]: 1.015–1.192; *p* = 0.020). Independent associations with AS were also observed for age (OR, 1.068 per year; 95% CI, 1.026–1.111; *p* = 0.001) and SBP (OR, 1.158 per mmHg; 95% CI, 1.098–1.221; *p* < 0.001). In the multivariate model, diabetes mellitus, fasting glucose, and UPCR were no longer statistically significant (Table 3). Collinearity diagnostics did not suggest severe multicollinearity among the predictors in the original logistic regression model. Variance inflation factor values ranged from 1.047 to 2.482. All the variance inflation factor values were below 3. In the sensitivity analysis, additionally adjusted for eGFR, the adjusted odds ratio for serum galectin-3 was 1.113 per 1 ng/mL increase (95% CI, 1.024–1.211; *p* = 0.012). The full results of the eGFR-adjusted model are presented in Supplementary Table S1. In supplementary sequentially adjusted logistic regression analyses, serum galectin-3 remained positively associated with AS after adjustment for age and sex and subsequent addition of SBP, diabetes mellitus, fasting glucose, eGFR, and UPCR. Across the adjusted models, the odds ratios for galectin-3 ranged from 1.088 to 1.114 per 1 ng/mL increase, and all associations were statistically significant. In the model incorporating all of these covariates, the adjusted odds ratio was 1.114 (95% CI, 1.024–1.212; *p* = 0.012). The model specifications and results are presented in Supplementary Table S2.

Restricted cubic spline diagnostics identified departures from linearity in the log odds of AS for SBP (*p* = 0.014), UPCR (*p* < 0.001), and fasting glucose (*p* = 0.046) in the eGFR-adjusted model. The nonlinear components for galectin-3 and eGFR did not reach the nominal significance threshold (*p* = 0.055 for each). In an exploratory model simultaneously incorporating spline terms for galectin-3, SBP, eGFR, and UPCR, galectin-3 remained associated with AS in the joint test of its linear and nonlinear components (overall *p* = 0.036). Detailed specifications and results are presented in Supplementary Table S3.

In the pooled sensitivity model additionally adjusted for CKD etiology, serum galectin-3 remained associated with AS (adjusted OR, 1.101 per 1 ng/mL increase; 95% CI, 1.008–1.203; *p* = 0.032). The model included age, sex, SBP, diabetes mellitus, fasting glucose, eGFR, and UPCR, together with CKD etiology. In the alternative etiology-adjusted model without a separate diabetes mellitus term, the adjusted OR for galectin-3 was 1.104 (95% CI, 1.011–1.206; *p* = 0.028). Both models included all 176 participants. Complete results are provided in Supplementary Table S4.

### 3.3. Determinants of cfPWV

Higher cfPWV was significantly associated with older age (*r* = 0.311, *p* < 0.001), higher SBP (*r* = 0.582, *p* < 0.001), higher log-transformed glucose (log-glucose, *r* = 0.252, *p* = 0.001), higher log-UPCR (*r* = 0.185, *p* = 0.014), lower eGFR (*r* = −0.166, *p* = 0.028), and higher serum log-transformed galectin-3 (*r* = 0.257, *p* = 0.001) in simple linear analysis. However, in the multivariable stepwise linear regression model, only age (β = 0.220; adjusted R^2^ change = 0.040, *p* < 0.001), SBP (β = 0.510; adjusted R^2^ change = 0.335, *p* < 0.001), and log-galectin-3 (β = 0.143; adjusted R^2^ change = 0.016, *p* = 0.020) remained independently associated with cfPWV (Table 4). The stepwise model explained 40.2% of the variability in cfPWV (R^2^ = 0.402; adjusted R^2^ = 0.391; model *p* < 0.001).

In the supplementary cfPWV model retaining all six continuous candidate predictors, restricted cubic spline analyses identified nonlinearity for ln(galectin-3) (*p* = 0.004) and SBP (*p* = 0.003), but not for eGFR (*p* = 0.320) or ln (UPCR) (*p* = 0.115). In the exploratory model incorporating spline terms for all four of these predictors simultaneously, the overall association between galectin-3 and cfPWV remained statistically significant (overall *p* = 0.008), with evidence of nonlinearity on the logarithmic galectin-3 scale (*p* = 0.007). These findings indicate that a single linear coefficient does not fully characterize the association (Supplementary Table S3).

### 3.4. Stage-Stratified Analyses

We evaluated potential differences in the association between log-galectin-3 and cfPWV using separate multivariate linear regression models for CKD stages 3, 4, and 5. In stage 3 CKD, cfPWV was independently associated with age (B = 0.055, beta = 0.233, *p* = 0.021) and SBP (B = 0.089, beta = 0.497; *p* < 0.001). In stage 4 CKD, the same variables remained significant, including age (B = 0.060, beta = 0.264, *p* = 0.015) and SBP (B = 0.106, beta = 0.505; *p* < 0·001).

In stage 5 CKD, SBP remained independently associated with cfPWV (B = 0.069, beta = 0.335, *p* = 0.027), whereas log-galectin-3 was also significantly associated with cfPWV (B = 7.353, beta = 0.394, *p* = 0.006). Age showed a borderline association (B = 0.049; beta = 0.251; *p* = 0.054).

All stage-specific models were statistically significant (all model *p* < 0.001). The R^2^ ranged from 0.420 to 0.527, while adjusted R^2^ values ranged from 0.368 to 0.467 (stage 3: 0.387; stage 4: 0.368; stage 5: 0.467). Overall, the selected covariates explained approximately 37–47% of the variability in cfPWV across CKD stages. The stage 5 model demonstrated the highest explanatory power (R^2^ = 0.527; adjusted R^2^ = 0.467).

Collectively, these findings suggest that determinants of cfPWV may differ across CKD stages, and that galectin-3 appears to be more strongly associated with cfPWV in advanced CKD (Table 5 and Table 6).

### 3.5. Correlates of Galectin-3

Spearman’s correlation analysis demonstrated that serum galectin-3 levels were significantly and positively correlated with SBP (ρ = 0.194, *p* = 0.010), total cholesterol (ρ = 0.176, *p* = 0.019), LDL-C (ρ = 0.166, *p* = 0.028), BUN (ρ = 0.182, *p* = 0.015), creatinine (ρ = 0.237, *p* = 0.002), UPCR (ρ = 0.394, *p* < 0.001), and cfPWV (ρ = 0.273, *p* < 0.001). In contrast, galectin-3 levels were significantly and negatively correlated with eGFR (ρ = −0.241, *p* = 0.001) (Table 7).

## 4. Discussion

The main contribution of this study is not the identification of galectin-3 as a general vascular biomarker, which has been suggested previously, but the evaluation of this association in a non-dialysis CKD cohort across stages 3–5 using cfPWV-defined AS. In addition, the association between galectin-3 and AS remained significant after adjustment for established cardiovascular and renal risk factors, suggesting that galectin-3 may capture fibro-inflammatory or cardiorenal-metabolic information not fully represented by conventional variables in this cohort.

The present study yielded several key findings. First, serum galectin-3 was independently associated with AS in patients with nondialysis CKD, and this association remained significant after adjustment for conventional clinical variables. Second, in multivariate linear regression analyses treating cfPWV as a continuous outcome, age, SBP, and log-galectin-3 were independently associated with cfPWV. Third, in stage-stratified analyses, the association between galectin-3 and cfPWV was more evident in patients with stage 5 CKD. Although serum galectin-3 was independently associated with AS in the overall cohort, the stage-specific association with cfPWV was statistically significant only in the stage 5 subgroup. The lack of statistical significance in stages 3 and 4 should not be interpreted as definitive evidence of no association, given the smaller subgroup sample sizes and the resulting limited statistical power. These findings should therefore be considered exploratory and suggest, rather than establish, that the relationship between galectin-3 and vascular stiffness may be more pronounced in advanced CKD. Larger studies incorporating formal interaction analyses are needed to determine whether CKD stage modifies this association. Given the weak-to-low correlation coefficients observed in the bivariate analyses and the absence of a significant stage-specific association in CKD stages 3 and 4, galectin-3 should not be interpreted as a strong standalone biomarker for arterial stiffness in moderate CKD. Rather, these findings support its further evaluation as one component of a broader cardiovascular risk-assessment framework that also includes established clinical determinants such as age and SBP.

Among these findings, the independent association between galectin-3 and cfPWV-defined AS requires clinical contextualization. In the multivariable logistic regression model, each 1 ng/mL increase in serum galectin-3 was associated with 10.0% higher odds of AS. In the linear regression analysis, log-transformed galectin-3 also remained independently associated with continuous cfPWV, although its incremental explanatory contribution was limited, with an adjusted R^2^ change of 0.016. These findings indicate that galectin-3 was associated with AS when cfPWV was analyzed either as a categorical outcome or as a continuous measure. However, because age and SBP accounted for a larger proportion of cfPWV variability, the clinical magnitude of the galectin-3 association should be interpreted as an additional biomarker signal rather than as a dominant determinant of AS. Galectin-3 is increasingly recognized as a mediator of inflammation, fibrosis, extracellular matrix remodeling, and tissue repair processes, all of which are closely linked to vascular stiffening [18,27]. Experimental and translational studies have shown that galectin-3 is involved in macrophage activation, oxidative stress, fibroblast signaling, and collagen deposition, mechanisms that may reduce arterial compliance and participate in vascular remodeling [18,28,29]. In the setting of CKD, chronic low-grade inflammation, uremic toxin accumulation, and reduced renal clearance may amplify these effects, creating a biological environment associated with both increased circulating galectin-3 levels and progressive vascular injury [30,31]. Because of the cross-sectional design of the present study, however, these findings cannot determine whether elevated galectin-3 precedes AS, results from related CKD-associated disease processes, or reflects common upstream determinants. Therefore, galectin-3 should be interpreted as a biomarker associated with AS rather than as evidence of a causal pathway in this cohort.

Our findings, together with prior studies, suggest that galectin-3 may provide pathophysiological information relevant to cardiovascular risk in CKD, particularly because it appears to reflect integrated fibro-inflammatory and cardiorenal-metabolic burden. However, because the present study evaluated associations with AS rather than incremental predictive performance, discrimination, calibration, or clinical reclassification, further prospective studies are needed before its role in cardiovascular risk assessment can be defined. In cardiovascular disease and atherosclerosis, galectin-3 is associated with macrophage activation, endothelial dysfunction, and fibro-inflammatory vascular remodeling, and these mechanisms may contribute to plaque growth or adverse cardiovascular risk [32]. However, in the kidney, its role seems to be dependent on different contexts. The review literature suggests that, while more consistently associated with inflammation, fibrosis, and CKD progression, some putative protective or reparative effects have been proposed in experimental studies of acute kidney injury and injury-repair settings [31,33]. Nevertheless, currently available human evidence supports its prognostic significance in CKD more strongly than it supports a definitive regenerative role in renal tissue [30]. This broader perspective is consistent with the review by Provenzano et al., which places galectin-3 within a multifaceted CKD-related cardiovascular biomarker network rather than as a standalone risk classifier [23]. Their synthesis also highlights that no single biomarker currently provides comprehensive cardiovascular risk prediction in CKD and that multimarker approaches with prospective validation will likely be needed before biomarker-guided clinical implementation can be recommended [23]. These findings should therefore be viewed as hypothesis-generating and as supportive of further evaluation of galectin-3 within a multimarker cardiovascular risk framework rather than as evidence that galectin-3 alone is ready for clinical risk stratification.

Our results are broadly consistent with prior studies in dialysis populations and extend those observations to earlier stages of kidney disease. In a previously published cohort of patients receiving peritoneal dialysis, serum galectin-3 was positively and independently associated with cfPWV and AS, with comparable predictive performance in receiver operating characteristic analyses [25]. These current findings replicate this association in a nondialysis CKD population, suggesting that the galectin-3–vascular stiffness relationship may be present before the initiation of dialysis. This is clinically important, as subclinical vascular injury occurs early in CKD and contributes substantially to heightened cardiovascular risk observed in this population, as highlighted in prior reviews and guidelines [1,7,11]. Overall, these data suggest that galectin-3 is not restricted to end-stage kidney disease but may reflect a continuum of cardiovascular risk across the CKD spectrum.

Age and SBP were the other two variables consistently and independently associated with AS in multivariable models. This pattern is biologically plausible and aligns with current concepts of vascular aging and pressure-mediated arterial remodeling. With aging, large arteries undergo elastin fragmentation, collagen accumulation, medial thickening, and vascular smooth muscle cell senescence, all of which contribute to increased stiffness [4,5,34]. Similarly, elevated SBP both reflects and promotes arterial stiffening through repetitive mechanical stress, endothelial dysfunction, and maladaptive structural remodeling of the vascular wall [35]. The consistent significance of age and SBP across CKD stages 3 and 4 highlights the dominant role of hemodynamic load and vascular aging in earlier CKD stages, prior to the more pronounced metabolic and uremic disturbances seen in advanced disease.

Patients with AS had a higher prevalence of diabetes mellitus and higher fasting glucose levels than those without AS. This finding is clinically plausible, as diabetic macroangiopathy and chronic hyperglycemia can promote arterial stiffening through the accumulation of advanced glycation end products, oxidative stress, endothelial dysfunction, inflammation, medial calcification, and vascular remodeling [9,13]. Galectin-3 concentrations may also be elevated in patients with diabetes and diabetic kidney disease, suggesting that galectin-3 may reflect an integrated fibro-inflammatory and cardiorenal-metabolic burden rather than an isolated vascular process. Nevertheless, several findings indicate that the association between galectin-3 and AS was not entirely explained by diabetes or current glycemic status. Although diabetes mellitus and fasting glucose were associated with AS or cfPWV in univariable analyses, neither remained statistically significant after multivariable adjustment. In contrast, serum galectin-3 remained independently associated with both categorical AS and continuous cfPWV. Furthermore, serum galectin-3 was not significantly correlated with fasting glucose. The attenuation of the associations of diabetes, fasting glucose, and UPCR after adjustment may partly reflect shared variance with more influential determinants, particularly age and SBP. These results therefore suggest that diabetes and hyperglycemia contribute to vascular injury but do not fully account for the observed relationship between galectin-3 and arterial stiffness.

The stage-stratified analyses further indicated that the determinants of cfPWV may vary according to CKD severity. In patients with stage 5 CKD, cfPWV remained independently associated with galectin-3, whereas the association with age was only borderline significant. This finding raises the possibility that fibro-inflammatory pathways, together with severe renal dysfunction and the accumulation of CKD-related metabolic disturbances, may have a greater role in vascular stiffening in advanced CKD. However, these stage-specific results should be interpreted cautiously because of the relatively small number of patients in each stratum.

Previous studies have reported elevated circulating galectin-3 levels in diabetes-related conditions, particularly diabetic nephropathy, as demonstrated in a systematic review and meta-analysis [36], as well as associations between galectin-3 and metabolic abnormalities in community-based populations [37]. Accordingly, galectin-3 may be more appropriately regarded as an integrated marker of fibrotic, inflammatory, renal, and cardiometabolic disease burden. Consistent with this interpretation, higher galectin-3 levels in our cohort were associated with higher BUN, creatinine, UPCR, total cholesterol, LDL-C, and cfPWV, and with lower eGFR. Previous studies have similarly linked galectin-3 to renal fibrosis, inflammatory activation, and CKD progression [21,30,31]. The observed associations with lipid parameters may indicate a broader relationship with cardiometabolic risk, although the evidence supporting these relationships remains limited and should be interpreted cautiously [38,39]. Recent histopathological evidence has further shown that dyslipidemic profiles, particularly LDL-C combined with remnant cholesterol or triglyceride abnormalities, are associated with carotid plaque instability, and that these associations may vary according to age and sex [40]. Therefore, the correlations between galectin-3, total cholesterol, and LDL-C observed in the present cohort may reflect a broader cardiometabolic vascular burden rather than a direct lipid-mediated pathway linking galectin-3 to AS.

Residual confounding cannot be excluded because information on diabetes duration, glycated hemoglobin, glycemic variability, diabetic macrovascular complications, and glucose-lowering therapies was not systematically collected. Therefore, the present findings should be interpreted as demonstrating an independent association between galectin-3 and AS rather than establishing a causal role for galectin-3 in the development of arterial stiffness.

Overall, our findings support an association between circulating galectin-3 and AS in adults with nondialysis CKD. The association with continuous cfPWV was more evident in stage 5 CKD; however, these exploratory subgroup findings do not establish that CKD stage modifies this relationship. Clinically, galectin-3 may be regarded as a pathophysiologically informative biomarker that potentially reflects overlapping fibrotic, inflammatory, and renal disease processes, rather than a validated standalone diagnostic tool. Several limitations warrant consideration. First, the cross-sectional design precludes conclusions regarding causality or the temporal relationship between galectin-3 and AS. The observed association may reflect shared underlying disease processes, and its direction cannot be determined. Second, galectin-3 was measured at a single time point, preventing assessment of within-person variability, longitudinal biomarker trajectories, and their relationship with changes in vascular stiffness. Third, covariate selection for the original logistic regression model was based on statistically significant between-group differences, which may have resulted in the omission of clinically relevant confounders. Because galectin-3 was inversely correlated with eGFR, confounding by kidney function was a particular concern. Additional sensitivity and sequentially adjusted analyses incorporated kidney function, demographic and cardiometabolic factors, proteinuria, and CKD etiology, and the galectin-3–AS association persisted across these specifications (Supplementary Tables S1, S2 and S4). Nevertheless, the limited number of AS cases relative to model complexity and the substantial overlap between diabetes mellitus and diabetic kidney disease constrained the precision of the expanded models. Medication use was not included in these adjusted models, and residual confounding by pharmacological treatment and other unmeasured or incompletely characterized factors remains possible. Accordingly, the findings should be interpreted as associations conditional on the included covariates, rather than evidence that galectin-3 is independent of the broader biological burden of CKD. Fourth, several potentially relevant biological pathways were not comprehensively assessed, including systemic inflammation, oxidative stress, mineral metabolism, uremic toxin burden, and cardiorenal interactions. In particular, intact parathyroid hormone, serum calcium, and serum phosphorus measurements were unavailable in the analytical dataset, precluding calculation of the calcium–phosphorus product and sensitivity analyses incorporating these parameters. Consequently, the contribution of CKD–mineral and bone disorder and associated vascular calcification to the observed association could not be determined. Adjustment for eGFR and CKD etiology does not substitute for direct assessment of mineral metabolism or establish independence from the bone–mineral–vascular calcification axis. Additional limitations concern the breadth of biomarkers and cardiovascular phenotyping. Established markers of kidney injury and repair, including neutrophil gelatinase-associated lipocalin, kidney injury molecule-1, fatty acid-binding protein, and osteopontin, were not measured because they were not prespecified in the original protocol and sufficient stored biological samples were unavailable for retrospective analysis. We therefore could not determine whether galec-tin-3 provides independent or complementary information beyond markers of tubular injury, inflammation, and renal fibrosis. Furthermore, although cfPWV is an established reference standard for assessing AS, complementary echocardiographic measures, including indices of aortic elasticity and left ventricular diastolic function, were not included in the protocol. More detailed characterization of cardiac function and local aortic properties was therefore unavailable. The modeling strategy also warrants caution. Forward-stepwise selection in the original cfPWV analysis may increase the risk of unstable variable selection, inflated type I error, and overestimated regression coefficients. Although exploratory restricted cubic spline analyses supported an overall association between galectin-3 and AS or cfPWV, they also identified departures from the originally specified linear functional forms for several predictors (Supplementary Table S3). The original per-unit odds ratios and regression coefficients should therefore not be interpreted as uniform associations across the entire observed predictor range. Moreover, the supplementary analyses were not adjusted for multiple testing, and the spline assessments did not establish the functional forms within individual CKD-stage subgroups or validate those retained in the subsequent etiology-adjusted models. These findings should consequently be considered exploratory and hypothesis-generating rather than confirmatory. Future studies should include larger, multicenter cohorts with longitudinal follow-up, repeated galectin-3 measurements, comprehensive clinical and treatment data, and multimarker panels encompassing inflammatory, fibrotic, endothelial, and kidney injury pathways. Incorporating mineral-metabolism measurements, direct assessments of vascular calcification, and complementary cardiac imaging would help clarify the biological processes underlying the observed association. Prespecified multivariable models, formal evaluation of differences across CKD stages and etiologies, and independent validation are needed to establish the reproducibility and incremental clinical value of galectin-3. Prospective studies should determine whether galectin-3 adds prognostic information beyond established cardiovascular and renal variables, whereas appropriately designed interventional studies would be required to evaluate whether targeting galectin-3-related pathways modifies CKD-associated vascular stiffening.

## 5. Conclusions

Higher serum galectin-3 levels were independently associated with categorical AS in the overall cohort. In an exploratory forward-stepwise analysis, log-galectin-3 was also retained as a factor associated with continuous cfPWV. In stage-stratified analyses, this association reached statistical significance only in patients with stage 5 CKD. These subgroup findings should be interpreted cautiously because of the limited sample size and require confirmation in larger prospective studies. Further longitudinal studies are warranted to clarify the clinical and prognostic significance of this biomarker.

## Figures and Tables

**Table 1:** Eligibility criteria for enrollment of non-dialysis patients with stage 3–5 CKD.

Item	Description
Inclusion criteria	Age ≥ 18 years; stage 3–5 CKD managed in the nephrology outpatient clinic; no maintenance dialysis at enrollment; CKD confirmed by repeated eGFR assessment over ≥3 months using the 2021 CKD-EPI equation.
Exclusion criteria	Active malignancy, chronic inflammatory disease, acute decompensated heart failure, acute coronary syndrome or another recent major cardiovascular event, chronic obstructive pulmonary disease, maintenance dialysis, planned kidney transplantation within the following 6 months, or refusal/inability to provide informed consent.

Abbreviations: CKD, chronic kidney disease; eGFR, estimated glomerular filtration rate; CKD-EPI, Chronic Kidney Disease Epidemiology Collaboration.

**Table 2:** Demographic, clinical, and laboratory characteristics stratified by aortic stiffness status.

Variables	Overall (*n* = 176)	No AS (*n* = 113)	AS (*n* = 63)	*p* Value
Age (years)	67.72 ± 12.98	65.07 ± 13.50	72.46 ± 10.52	<0.001 *
Height (cm)	159.32 ± 8.95	159.64 ± 8.50	158.73 ± 9.74	0.518
Body weight (kg)	67.44 ± 15.20	66.99 ± 14.9	68.24 ± 15.79	0.600
Body mass index (kg/m^2^)	26.42 ± 4.71	26.13 ± 4.62	26.92 ± 4.85	0.289
cfPWV (m/s)	9.48 ± 2.85	7.74 ± 1.44	12.59 ± 1.96	<0.001 *
SBP (mmHg)	136.00 (131.00–146.75)	133.00 (127.00–138.00)	152.00 (139.00–158.00)	<0.001 *
DBP (mmHg)	83.67 ± 13.30	82.57 ± 10.89	85.65 ± 14.38	0.111
Hemoglobin (g/dL)	11.37 ± 1.96	11.37 ± 2.02	11.36 ± 1.86	0.964
Total cholesterol (mg/dL)	160.85 ± 40.06	158.18 ± 38.24	165.63 ± 43.04	0.237
Triglyceride (mg/dL)	127.0 (92.50–176.25)	120.00 (92.00–168.50)	140.00 (94.00–188.00)	0.293
LDL-C (mg/dL)	90.85 ± 34.51	88.61 ± 33.89	94.86 ± 35.52	0.251
Fasting glucose (mg/dL)	118.00 (98.00–138.00)	102.00 (97.00–129.50)	134.00 (120.00–155.00)	<0.001 *
Albumin (g/dL)	4.10 (3.90–4.30)	4.09 (3.90–4.25)	4.10 (3.90–4.30)	0.801
Blood urea nitrogen (mg/dL)	38.50 (26.25–53.75)	38.00 (26.00–56.00)	40.00 (30.00–51.00)	0.463
Creatinine (mg/dL)	2.20 (1.60–3.38)	2.20 (1.60–3.35)	2.2 (1.60–3.50)	0.603
eGFR (mL/min/1.73 m^2^)	28.19 ± 15.02	29.28 ± 15.77	26.24 ± 13.46	0.200
Spot UPCR (g/g)	0.27 (0.14–0.74)	0.21 (0.11–0.73)	0.32 (0.22–0.74)	0.006 *
Galectin-3 (ng/mL)	14.47 (11.92–20.38)	13.75 (11.70–17.82)	18.33 (12.24–23.83)	0.002 *
Female, *n* (%)	73 (41.5)	49 (43.4)	24 (38.1)	0.497
Diabetes mellitus, *n* (%)	71 (40.3)	33 (29.2)	38 (60.3)	<0.001 *
Hypertension, *n* (%)	144 (81.8)	95 (84.1)	49 (77.8)	0.299
ARB use, *n* (%)	100 (56.8)	64 (56.6)	36 (57.1)	0.948
β-blocker use, *n* (%)	52 (29.5)	33 (29.2)	19 (30.2)	0.894
CCB use, *n* (%)	92 (52.3)	57 (50.4)	35 (55.6)	0.515
Statin use, *n* (%)	82 (46.6)	51 (45.1)	31 (49.2)	0.604
Fibrate use, *n* (%)	51 (29.0)	33 (29.2)	18 (28.6)	0.929
CKD stage 3, *n* (%)	70 (39.8)	48 (42.5)	22 (34.9)	0.523
CKD stage 4, *n* (%)	61 (34.7)	36 (31.9)	25 (39.7)	
CKD stage 5, *n* (%)	45 (25.6)	29 (25.7)	16 (25.4)	
Cause of CKD				
Diabetic kidney disease, *n* (%)	67 (38.1)	32 (28.3)	35 (55.6)	<0.001 *
Glomerulonephritis, *n* (%)	46 (26.1)	33 (29.2)	13 (20.6)	0.215
Hypertensive nephrosclerosis, *n* (%)	25 (14.2)	19 (16.8)	6 (9.5)	0.184
Other or unknown, *n* (%)	38 (21.6)	29 (25.7)	9 (14.3)	0.079

Values are presented as mean ± standard deviation, median (interquartile range), or number (%), as appropriate. Comparisons between groups were performed using the independent Student’s *t*-test, Mann–Whitney U test, or chi-square test. AS was defined as cfPWV > 10 m/s. A two-sided *p*-value < 0.05 was considered statistically significant. Abbreviations: AS, aortic stiffness; cfPWV, carotid–femoral pulse wave velocity; SBP, systolic blood pressure; DBP, diastolic blood pressure; LDL-C, low-density lipoprotein cholesterol; eGFR, estimated glomerular filtration rate; UPCR, urine protein-to-creatinine ratio; ARB, angiotensin receptor blocker; CCB, calcium channel blocker; CKD, chronic kidney disease. * *p* < 0.05 indicates statistical significance.

**Table 3:** Multivariable logistic regression model for factors associated with aortic stiffness.

Variables	OR	95% CI	*p* Value	VIF
Serum galectin-3 (per 1 ng/mL)	1.100	1.015–1.192	0.020 *	1.285
Age (per 1 year)	1.068	1.026–1.111	0.001 *	1.047
Systolic blood pressure (per 1 mmHg)	1.158	1.098–1.221	<0.001 *	1.195
Diabetes mellitus (present)	2.062	0.547–7.771	0.285	2.482
Fasting glucose (per 1 mg/dL)	1.008	0.989–1.028	0.414	2.399
Spot UPCR (per 1 g/g)	0.779	0.302–1.805	0.507	1.215

Variables entered into the multivariable logistic regression model were diabetes mellitus, age, systolic blood pressure, fasting glucose, urine protein-to-creatinine ratio, and serum galectin-3. Abbreviations: OR, odds ratio; CI, confidence interval; VIF, variance inflation factor; UPCR, urine protein-to-creatinine ratio. * A *p*-value < 0.05 indicated statistical significance.

**Table 4:** Simple and multivariable linear regression analyses of factors associated with cfPWV.

Variables		Carotid-Femoral Pulse Wave Velocity (m/s)				
	Simple Correlation			Multivariable Linear Regression		
	*r*	*p* Value	B	β	Adjusted R^2^ Change	*p* Value
Age (years)	0.311	<0.001 *	0.048	0.220	0.040	<0.001 *
Height (cm)	−0.031	0.682	—	–	–	–
Body weight (kg)	0.032	0.669	—	–	–	–
Body mass index (kg/m^2^)	0.051	0.504	—	–	–	–
SBP (mmHg)	0.582	<0.001 *	0.098	0.510	0.335	<0.001 *
DBP (mmHg)	0.140	0.064	—	–	–	–
Hemoglobin (g/dL)	−0.105	0.167	—	–	–	–
Total cholesterol (mg/dL)	0.097	0.200	—	–	–	–
Log-Triglyceride (mg/dL)	0.081	0.288	—	–	–	–
LDL-C (mg/dL)	0.074	0.328	—	–	–	–
Log-Glucose (mg/dL)	0.252	0.001 *	—	–	–	–
Log-Albumin (g/dL)	0.026	0.734	—	–	–	–
Log-BUN (mg/dL)	0.094	0.214	—	–	–	–
Log-Creatinine (mg/dL)	0.116	0.127	—	–	–	–
eGFR (mL/min/1.73 m^2^)	−0.166	0.028 *	—	–	–	–
Log-UPCR (g/g)	0.185	0.014 *	—	–	–	–
Log-Galectin-3 (ng/mL)	0.257	0.001 *	0.953	0.143	0.016	0.020 *

Triglycerides, fasting glucose levels, albumin, BUN, creatinine, UPCR, and galectin-3 were log-transformed before analysis. Variables associated with cfPWV in simple analysis were subsequently entered into the multivariable stepwise linear regression model. *B* indicates the unstandardized regression coefficient, and standardized β indicates the standardized regression coefficient. Variables not retained in the stepwise model are indicated by em dashes. Abbreviations: cfPWV, carotid-femoral pulse wave velocity; SBP, systolic blood pressure; DBP, diastolic blood pressure; LDL-C, low-density lipoprotein cholesterol; BUN, blood urea nitrogen; eGFR, estimated glomerular filtration rate; UPCR, urine protein-to-creatinine ratio. Results with *p*-values < 0.05 were regarded as statistically significant. Model R^2^ = 0.402; adjusted R^2^ = 0.391; *p* < 0.001 *.

**Table 5:** Stage-specific multivariate linear regression analyses of factors associated with cfPWV.

CKD Stage	Predictor	B	SE	β	t	95% CI	*p* Value
Stage 3	Age	0.055	0.023	0.233	2.376	0.009–0.101	0.021 *
	SBP	0.089	0.018	0.497	4.908	0.053–0.125	<0.001 *
	Log-glucose	4.474	2.441	0.183	1.833	−0.402–9.350	0.071
	Log-UPCR	0.900	0.634	0.150	1.418	−0.368–2.167	0.161
	Log-Galectin-3	−1.105	1.559	−0.076	−0.709	−4.218–2.009	0.481
Stage 4	Age	0.060	0.024	0.264	2.513	0.012–0.108	0.015 *
	SBP	0.106	0.023	0.505	4.727	0.061–0.152	<0.001 *
	Log-glucose	2.000	3.036	0.073	0.659	−4.085–8.085	0.513
	Log-UPCR	0.599	0.713	0.095	0.840	−0.830–2.028	0.405
	Log-Galectin-3	2.302	1.707	0.150	1.349	−1.118–5.722	0.183
Stage 5	Age	0.049	0.025	0.251	1.986	−0.001–0.099	0.054
	SBP	0.069	0.030	0.335	2.294	0.008–0.130	0.027 *
	Log-glucose	3.894	3.616	0.132	1.077	−3.420–11.208	0.288
	Log-UPCR	−0.068	0.834	−0.011	−0.081	−1.755–1.619	0.936
	Log-Galectin-3	7.353	2.541	0.394	2.894	2.215–12.492	0.006 *

Analyses were stratified by CKD stage, and cfPWV was considered a continuous dependent variable. Separate multivariable linear regression models were fitted for CKD stages 3, 4, and 5. Fasting glucose, urine protein-to-creatinine ratio, and galectin-3 were analyzed on the logarithmic scale because of skewed distributions. B represents the unstandardized regression coefficient, and Beta represents the standardized regression coefficient. Abbreviations: cfPWV, carotid-femoral pulse wave velocity; CKD, chronic kidney disease; CI, confidence interval; SE, standard error. Results with *p* values < 0.05 were regarded as statistically significant. * *p* < 0.05 indicates statistical significance.

**Table 6:** Model performance of the stage-stratified multivariable linear regression analyses for cfPWV.

CKD Stage	*n*	df	R^2^	Adjusted R^2^	F Statistic	Model *p* Value	RMSE	AIC	BIC
Stage 3	70	64	0.431	0.387	9.695	<0.001 *	2.163	312.378	325.869
Stage 4	61	55	0.420	0.368	7.977	<0.001 *	2.276	279.131	291.796
Stage 5	45	39	0.527	0.467	8.701	<0.001 *	2.167	202.851	213.691

Model performance was evaluated for the stage-stratified multivariable linear regression models shown in Table 5 using R^2^, adjusted R^2^, the F statistic, model *p* value, root mean square error (RMSE), Akaike information criterion (AIC), and Bayesian information criterion (BIC). Abbreviations: df, residual degrees of freedom; RMSE, root mean square error; AIC, Akaike information criterion; BIC, Bayesian information criterion. * *p* < 0.05 indicates statistical significance.

**Table 7:** Spearman correlation analysis of serum galectin-3 and clinical variables.

Variables	Spearman Correlation Coefficient	*p* Value
Age (years)	0.008	0.914
Height (cm)	0.065	0.391
Body weight (kg)	0.032	0.671
Body mass index (kg/m^2^)	−0.008	0.920
SBP (mmHg)	0.194	0.010 *
DBP (mmHg)	0.106	0.161
Hemoglobin (g/dL)	−0.110	0.146
Total cholesterol (mg/dL)	0.176	0.019 *
Triglyceride (mg/dL)	0.025	0.745
LDL-C (mg/dL)	0.166	0.028 *
Glucose (mg/dL)	0.075	0.321
Albumin (g/dL)	−0.030	0.691
BUN (mg/dL)	0.182	0.015 *
Creatinine (mg/dL)	0.237	0.002 *
eGFR (mL/min/1.73 m^2^)	−0.241	0.001 *
UPCR (g/g)	0.394	<0.001 *
cfPWV (m/s)	0.273	<0.001 *

Abbreviations: SBP, systolic blood pressure; DBP, diastolic blood pressure; LDL-C, low-density lipoprotein cholesterol; BUN, blood urea nitrogen; eGFR, estimated glomerular filtration rate; UPCR, urine protein-to-creatinine ratio; cfPWV, carotid-femoral pulse wave velocity. * *p* < 0.05 indicates statistical significance.

## Data Availability

The datasets generated and/or analyzed in the present study are available from the corresponding author upon reasonable request.

## Supplementary Materials

The following supporting information can be downloaded at: https://www.mdpi.com/article/10.3390/biomedicines14102213/s1, Table S1: Multivariable logistic regression analysis of factors associated with aortic stiffness with sensitivity analysis incorporating estimated glomerular filtration rate in 176 patients with chronic kidney disease; Table S2: Sequentially adjusted logistic regression models for the association of serum galectin-3 with aortic stiffness in adults with stage 3–5 non-dialysis chronic kidney disease; Table S3. Restricted cubic spline assessment of continuous-variable functional forms; Table S4. CKD etiology-adjusted sensitivity analyses of the association between serum galectin-3 and aortic stiffness.

## Abbreviations

The following abbreviations are used in this manuscript:

AS	Aortic stiffness
BUN	Blood urea nitrogen
CI	Confidence interval
CKD	Chronic kidney disease
CKD-EPI	Chronic Kidney Disease Epidemiology Collaboration
cfPWV	Carotid-femoral pulse wave velocity
DBP	Diastolic blood pressure
eGFR	Estimated glomerular filtration rate
LDL-C	Low-density lipoprotein cholesterol
OR	Odds ratio
PWV	Pulse wave velocity
SBP	Systolic blood pressure
UPCR	Urine protein-to-creatinine ratio
