# Supplementary material for: Association of Serum Galectin-3 with Aortic Stiffness in Adults with Stage 3–5 Non-Dialysis Chronic Kidney Disease: Overall and Stage-Stratified Analyses
Source: Biomedicines. 2026 Sep 30;14(10):2213. doi: 10.3390/biomedicines14102213 (PMC13701507; doi:10.3390/biomedicines14102213)
Supplement: Supplementary file 1 [file biomedicines-14-02213-s001.zip › biomedicines-4519489-supplementary.pdf]

Supplementary Table S1. Multivariable logistic regression analysis of factors associated with aortic stiffness with sensitivity analysis incorporating estimated glomerular filtration rate in 176 patients with chronic kidney disease.

Variables	Odds ratio	95% confidence interval	*p* Value	VIF
Serum galectin-3, per 1 ng/mL	1.113	1.024–1.211	0.012*	1.364
Age, per 1 year	1.069	1.026–1.114	0.001*	1.048
Systolic blood pressure, per 1 mmHg	1.164	1.103–1.229	<0.001*	1.246
Diabetes mellitus, present vs. absent	2.059	0.545–7.773	0.287	2.483
Fasting glucose, per 1 mg/dL	1.007	0.988–1.027	0.465	2.426
Spot UPCR, per 1 g/g	0.708	0.293–1.712	0.443	1.218
eGFR, per 1 mL/min/1.73 m²	1.019	0.985–1.054	0.287	1.153

All seven predictors were entered simultaneously into the multivariable logistic regression model: eGFR, diabetes mellitus, age, systolic blood pressure, fasting glucose, UPCR, and serum galectin-3. Aortic stiffness was defined as cfPWV >10 m/s. The analysis included 176 patients (63 with aortic stiffness and 113 without). There were no missing values for the outcome or model covariates; no observations were excluded from this analysis, and no imputation was performed. Adjusted odds ratios were estimated by maximum likelihood, with Wald 95% confidence intervals and two-sided Wald tests. Abbreviations: cfPWV, carotid–femoral pulse wave velocity; eGFR, estimated glomerular filtration rate; UPCR, urine protein-to-creatinine ratio; VIF, variance inflation factor. *$p < 0.05$ was considered statistically significant.

Supplementary Table S2. Sequentially adjusted logistic regression models for the association of serum galectin-3 with aortic stiffness in adults with stage 3–5 non-dialysis chronic kidney disease.

**Model**	**Adjustment covariates**	**OR**	**95% CI**	***p* Value**
Unadjusted	None	1.089	1.037–1.143	<0.001*
Model 1	Age and sex	1.098	1.042–1.156	<0.001*
Model 2	Model 1 + systolic blood pressure, diabetes mellitus, and fasting glucose	1.088	1.015–1.167	0.017*
Model 3	Model 2 + eGFR	1.097	1.021–1.179	0.012*
Model 4	Model 3 + UPCR	1.114	1.024–1.212	0.012*

All odds ratios represent the association with a 1 ng/mL increase in serum galectin-3. Adjustment was cumulative: each model retained all covariates from the preceding model. Galectin-3 was included in every model. All models included 176 participants (63 with AS and 113 without AS). AS was defined as carotid–femoral pulse wave velocity >10 m/s. No values were missing for the outcome or the variables included in these models; no imputation was performed. Binary logistic regression was fitted by maximum likelihood with an intercept and forced entry of the specified predictors. Continuous variables were entered on their original scales. Confidence intervals and two-sided *p*-values were obtained using Wald inference. All five models converged. The maximum VIF across predictors in the adjusted models was 2.496. Abbreviations: AS, aortic stiffness; OR, odds ratio; CI, confidence interval; eGFR, estimated glomerular filtration rate; UPCR, urine protein-to-creatinine ratio; VIF, variance inflation factor. *$p < 0.05$ was considered statistically significant.

Supplementary Table S3. Restricted cubic spline assessment of continuous-variable functional forms.

A. Logistic regression for aortic stiffness

Predictor / modeled scale	Knots: 10th; 50th; 90th percentiles	Original model p for nonlinearity	eGFR-adjusted model *p* for nonlinearity
Serum galectin-3 (ng/mL)	9.0599; 14.4664; 26.2323	0.069	0.055
SBP (mmHg)	119.0000; 136.0000; 158.0000	0.017*	0.014*
eGFR (mL/min/1.73 m²)	10.3500; 25.3500; 51.5000	Not included	0.055
UPCR (g/g)	0.0900; 0.2745; 1.3326	0.001*	<0.001*
Age (years)	51.0000; 67.0000; 84.0000	0.465	0.547
Fasting glucose (mg/dL)	94.0000; 118.0000; 160.5000	0.034*	0.046*

For each continuous predictor separately, the existing linear term was retained, and one nonlinear restricted cubic spline component was added. Three knots were set at the empirical 10th, 50th, and 90th percentiles of the modeled predictor. All other covariates and their functional forms were held unchanged. Nested likelihood-ratio tests compared the augmented spline model with its linear-term counterpart (1 additional degree of freedom). Each row, therefore, represents a separate model comparison, not a model with all predictors simultaneously fitted as splines. * $p < 0.05$. Tests are exploratory and were not adjusted for multiple comparisons. Knot values are rounded for display only; unrounded values were used for fitting. Abbreviations: AS, aortic stiffness; cfPWV, carotid–femoral pulse wave velocity; CKD, chronic kidney disease; eGFR, estimated glomerular filtration rate; SBP, systolic blood pressure; UPCR, urine protein-to-creatinine ratio.

B. Linear regression for continuous cfPWV

Predictor / modeled scale	Knots: 10th; 50th; 90th percentiles	Original final model *p* for nonlinearity	Full candidate model *p* for nonlinearity	Full candidate model HC3 *p*
ln(galectin-3)	2.2038; 2.6718; 3.2670	0.001*	0.004*	0.003*

SBP (mmHg)	119.0000; 136.0000; 158.0000	<0.001*	0.003*	0.002*
eGFR (mL/min/1.73 m^2)	10.3500; 25.3500; 51.5000	Not included	0.320	0.396
ln(UPCR)	-2.4080; -1.2928; 0.2872	Not included	0.115	0.119
Age (years)	51.0000; 67.0000; 84.0000	0.332	0.346	0.393
ln(fasting glucose)	4.5433; 4.7707; 5.0783	Not included	0.656	0.707

Each predictor was assessed separately by adding one nonlinear spline component to the corresponding baseline model, using three knots at the 10th, 50th, and 90th percentiles of its modeled scale. Partial F tests compared nested ordinary least-squares models (1 additional numerator degree of freedom). The original-final and full-candidate augmented models had 171 and 168 residual degrees of freedom, respectively. HC3 heteroscedasticity-robust t tests of the added nonlinear coefficient were also calculated for the full candidate model as a sensitivity check. * *p* <0.05. Abbreviations: HC3, heteroscedasticity-consistent covariance estimator, type 3. Tests are exploratory and unadjusted for multiplicity. Knot values are shown on the transformed scale where applicable; all logarithms in these supplementary analyses are natural logarithms.

C. Exploratory joint spline sensitivity analyses

**Predictor (AS / cfPWV scale)**	**AS overall *p***	**AS nonlinear *p***	**cfPWV overall *p***	**cfPWV nonlinear *p***
Galectin-3 / ln(galectin-3)	0.036*	0.155	0.008*	0.007*
SBP	<0.001*	0.014*	<0.001*	0.008*
eGFR	0.101	0.101	0.365	0.163
UPCR / ln(UPCR)	0.026*	0.009*	0.366	0.200

cfPWV was regressed on spline terms for ln(galectin-3), SBP, eGFR, and ln(UPCR), together with linear terms for age and ln(fasting glucose). This model included 10 predictor parameters plus an intercept (176 participants; 165 residual degrees of freedom). Knot locations matched panel B. The joint partial F test for adding all four nonlinear components had 4 numerator degrees of freedom (*p* <0.001). * *p* <0.05. Abbreviations: AS, aortic stiffness; cfPWV, carotid–femoral pulse wave velocity; eGFR, estimated glomerular filtration rate; SBP, systolic blood pressure; UPCR, urine protein-to-creatinine ratio.

Supplementary Table S4. CKD etiology-adjusted sensitivity analyses of the association between serum galectin-3 and aortic stiffness.

**Model**	**Adjusted OR**	**95% CI**	***p* Value**
Model A: without CKD etiology	1.114	1.024–1.212	0.012*
Model B: with CKD etiology and DM	1.101	1.008–1.203	0.032*
Model C: with CKD etiology, without a separate DM term	1.104	1.011–1.206	0.028*

All estimates refer to a 1 ng/mL increase in serum galectin-3. Model A included galectin-3, age, sex, SBP, DM, fasting glucose, eGFR, and UPCR. Model B added the three CKD-etiology indicators to Model A. Model C retained all Model B terms except the separate DM term, as a secondary check prompted by the strong overlap between DM and DKD. All models used the same 176 participants, including 63 with AS and 113 without AS. AS was defined as cfPWV >10 m/s. Abbreviations: AS, aortic stiffness; cfPWV, carotid–femoral pulse wave velocity; CI, confidence interval; CKD, chronic kidney disease; DKD, diabetic kidney disease; DM, diabetes mellitus; eGFR, estimated glomerular filtration rate; OR, odds ratio; SBP, systolic blood pressure; UPCR, urine protein-to-creatinine ratio; VIF, variance inflation factor. *$p < 0.05$; two-sided Wald tests.
